# Pharmacokinetic Profiling and Simultaneous Determination of Thiopurine Immunosuppressants and Folic Acid by Chromatographic Methods

**DOI:** 10.3390/molecules24193469

**Published:** 2019-09-24

**Authors:** Edvin Brusač, Mario-Livio Jeličić, Daniela Amidžić Klarić, Biljana Nigović, Nikša Turk, Ilija Klarić, Ana Mornar

**Affiliations:** 1 Faculty of Pharmacy and Biochemistry, University of Zagreb, A. Kovačića 1, 10000 Zagreb, Croatia; ebrusac@pharma.hr (E.B.); mljelicic@pharma.hr (M.-L.J.); damidzic@pharma.hr (D.A.K.); bnigovic@pharma.hr (B.N.); 2 Clinical Hospital Center Zagreb, Kišpatićeva 12, 10000 Zagreb, Croatia; niksa_turk@net.hr; 3 Public Health Brčko DC, R. Dž. Čauševića 1, 76100 Brčko DC, Bosnia and Herzegovina; klaric67@gmail.com

**Keywords:** inflammatory bowel disease, fixed-dose combination, biomimetic chromatography, thiopurine immunosuppressants, folic acid

## Abstract

With the increase in the number of medicines patients have to take, there has been a rapid rise of fixed-dose combinations (FDCs) in the last two decades. Prior to FDC development, pharmacokinetic properties of active pharmaceutical ingredients (APIs) have to be evaluated, as well as methods for their determination developed. So as to increase patient compliance in inflammatory bowel disease, three novel FDCs of thiopurine immunosuppressants and folic acid are proposed; physico-chemical and pharmacokinetic properties such as hydrophobicity, lipophilicity and plasma protein binding of all APIs are evaluated. Moreover, experimental results of different properties are compared to those computed by various on-line prediction platforms so as to evaluate the viability of the in silico approach. A simultaneous method for their determination is developed, optimized, validated and applied to commercial tablet formulations. The method has shown to be fast, selective, accurate and precise, showing potential for reliable determination of API content in proposed FDCs during its development.

## 1. Introduction

Inflammatory bowel disease (IBD) comprises a group of idiopathic inflammatory gastrointestinal diseases with intestinal and extraintestinal manifestations. Crohn’s disease (CD) and ulcerative colitis (UC) are two main phenotypes of IBD; conditions that are chronic and often progressive with periods of exacerbation and remission. Despite the wide range of biological agents arising in IBD each year, the cornerstone of IBD treatment are still classical immunosuppressants (methotrexate, thiopurines), corticosteroids in flares and 5-aminosalicylates predominantly in UC [1]. Interventions with antibiotics, such as ciprofloxacin and corticosteroids, are often required in addition to maintenance therapy [2]. Moreover, IBD patients are at a risk for a variety of nutritional deficiencies because of reduced nutrient intake or absorption, as well as increased nutrient losses. Numerous nutrient deficiencies have been reported in IBD patients with varying degrees of prevalence and clinical significance. Many studies have shown that folate deficiency is one of the most noteworthy in CD and UC patients. It is common in up to 80% of IBD patients and is associated with megaloblastic anemia and increased risk of developing colorectal cancer [3,4,5]. Moreover, folic acid, according to European Crohn and Colitis Organization (ECCO) guidelines, should be recommended in all IBD patients in anticipation of pregnancy. Several studies have shown that some patients need to be on even higher doses of folic acid than the ECCO recommended dose [6].

Medication compliance has shown to be an important challenge in the treatment of IBD patients as the effectiveness of therapy requires long-term management to induce and maintain clinical remission. Some studies have shown that medication noncompliance in IBD patients is extremely high (up to 60% of patients) [7]. Compliance rates are particularly low among IBD patients during disease remission. During this period, patients often forget or deliberately avoid taking medications at the scheduled time [7,8].

For diseases that require treatment with multiple drugs, such as CD and UC, fixed-dose combination (FDC) therapy, in which two or more active pharmaceutical ingredients (APIs) are formulated in a fixed proportional manner into a single dosage form, may offer help in addressing some of the problems of compliance. Strategies for development of FDCs are complex and primarily based on fundamental understanding of therapeutic effect of drug combinations, clinical experience, drug-drug interactions, drug-excipient interactions, pharmacokinetic profiles and formulation challenges [9,10]. Although the number of the marketed FDC products has grown over the years and the trend is likely to continue, development of these products is quite demanding. The reason for such high complexity lies in the fact that the majority of FDC products contain two or more APIs with different physico-chemical and biopharmaceutical properties. To cope with this issue, the rational strategy for development of a new FDC product includes evaluation of pharmacokinetic profiles of APIs as well as development of a simultaneous analytical method for determination of APIs in early phases of FDC development [9,11].

**Figure 1:**
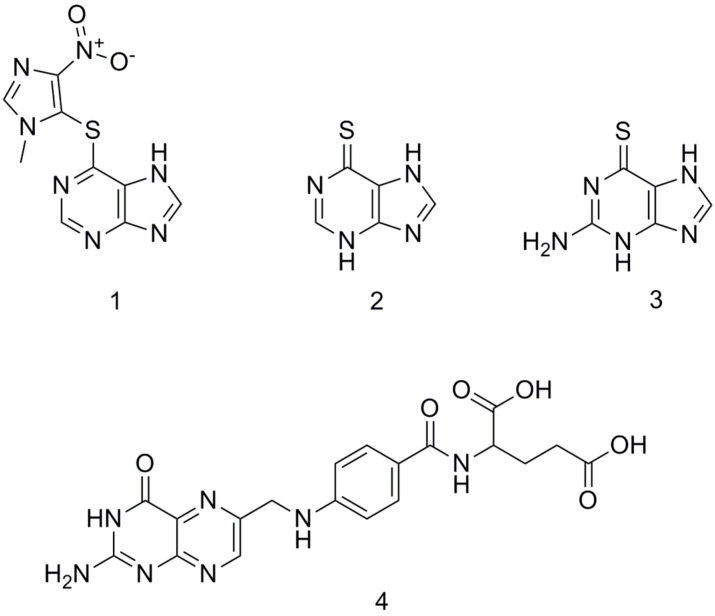
Chemical structures of azathioprine (**1**), 6-mercaptopurine (**2**), 6-thioguanine (**3**) and folic acid (**4**).

A vast majority of IBD patients are effectively treated with thiopurine immunosuppressants (azathioprine, 6-mercaptopurine and 6-thioguanine) as steroid-sparing therapy in combination with folic acid to improve the outcomes of medical treatment (Figure 1). Still, due to limited research and development of FDC in IBD, these products are not yet available at the market.

Biomimetic chromatography has unfolded new perspectives for the use of chromatographic techniques in drug development as it provides simple, reliable and inexpensive measurements of drug affinity to phospholipids and human proteins. The availability of this data in early stages of FDC development is crucial for rational selection of drug combination, their pharmacokinetic profiling and, finally, formulation development. So far, to our knowledge, limited data on biomimetic chromatographic behavior of thiopurine immunosuppressants and folic acid are available in literature [12].

Simultaneous analytical methods for determination of active ingredients in FDC should be developed in advance, since these methods are required for finished product quality control monitoring and dissolution testing. The review of literature reveals that there are many analytical methods for determination of immunosuppressants and folic acid individually in pharmaceutical dosage forms. In the last decade, various techniques for determination of azathioprine have been used, including high performance thin-layer chromatography [13], spectrophotometry [14], atomic absorption spectrometry [15], flow injection chemiluminometry [16], electrochemistry [17,18,19] and high-performance liquid chromatography [20]. Several methods have been reported for determination of 6-mercaptopurine in pharmaceutical formulations using analytical techniques such as Raman spectroscopy [21], electrochemistry [22] and high-performance liquid chromatography [23]. In literature, several analytical methods to quantify 6-thioguanine using fluorescence spectroscopy [24], electrochemistry [25] and high performance liquid chromatography with post column iodine-azide reaction [26] can be found. Many methods for determination of folic acid have been developed due to its biological significance. These methods include fluorescence spectroscopy [27]; spectrophotometry [28]; electrochemistry [29,30,31]; high-performance liquid chromatography coupled with various detectors, such as diode-array detector (DAD) [32]; tandem mass spectrometry [33] and corona-charged aerosol [34] detector and ultra-performance liquid chromatography [35]. Determination of these compounds in combination with other drugs by various techniques has been reported. The most commonly used technique is electrochemistry [36,37,38,39], while less prevalent are high-performance liquid chromatography [40] and electrophoresis [41]. To the best of our knowledge, no method for simultaneous determination of all these compounds was proposed. 

In light of the foregoing considerations, in this work pharmacokinetic properties of thiopurine immunosuppressants and folic acid were systematically evaluated using biomimetic chromatography. In addition, an analytical method for their simultaneous determination in proposed dose ratio as a prerequisite for development of three FDCs containing folic acid and one of thiopurine immunosuppressants is proposed.

## 2. Results and Discussion

### 2.1. Pharmacokinetic Profiling

#### 2.1.1. RP-TLC and RP-HPLC Assays

In reversed-phase chromatography, retention of analytes is governed by hydrophobicity, and this parameter was evaluated using both TLC and HPLC techniques. Due to high affinity of investigated compounds for octadecylsilane chemically bonded to porous silica applied on chromatographic plates, several analyses with mixtures of phosphate-buffered saline with various concentrations of methanol as mobile phase were performed. Methanol was chosen as the most suitable organic modifier, since it does not disturb the hydrogen bonding network of water. Although TLC technique has limited automation capabilities compared to other chromatographic techniques, its major advantage is the possibility for simultaneous determination of hydrophobicity of several compounds on the same chromatographic plate. *R*_M_ values were obtained according to Equation (1):

(1) $$R_{M}=\log\left(\frac{1}{R_{F}}-1\right),$$

where *R*_F_ represents the retention factor. Furthermore, chromatographic parameter of hydrophobicity, *R*_Mw_, was obtained by extrapolation to pure buffer using the linear Equation (2):

(2) $$R_{M}\;=\;R_{\mathrm{Mw}}\;-\;S\Phi ,$$

where S is the slope of the regression line and φ is the concentration (expressed as volume fraction) of methanol. Linear relationships between *R*_M_ and φ values were found for all compounds in the range of eluent composition examined (*r* ≥ 0.98). All *R*_M_ values are the average of three measurements, with relative standard deviations (RSDs) lower than 4.5%; the 95% confidence interval associated with each *R*_Mw_ value never exceeded 0.16 (Table 1). 

Further to the results presented above, all investigated compounds were expected to show high affinity for chromatographic column packing consisting of octadecylsilane-silica particles. The obtained retention times, *t*_R_, were used to calculate log *k* values according to Equation (3):

(3) $$\log k=\log\frac{t_{R}-t_{0}}{t_{0}},$$

with *t*_0_ being the retention time of an unretained solute, sodium nitrate. Furthermore, chromatographic parameter of hydrophobicity, log *k*_w C18_, was obtained by extrapolation to pure buffer using the linear Equation (4):

(4) $$\log k\;=\;\log k_{w}\;-\;S\Phi ,$$

where S is the slope of the regression line and φ is the concentration (expressed as volume fraction) of methanol. Linear relationships between log *k* and φ values were found for all compounds in the range of eluent composition examined (*r* ≥ 0.99). Possible occurrence of retention changes due to column aging was monitored by checking the retention times of test compound (ibuprofen). During the study, retention time of test compound changed no more than 3.2%, and no correction was done to the retention times experimentally determined for the analytes. All log *k* values are the average of three measurements, with RSD values lower than 4.4%; the 95% confidence interval associated with each log *k*_w C18_ value never exceeded 0.25 (Table 1). 

The results indicate that both experimental chromatographic methods yielded similar data. Positive hydrophobicity parameters were found for all analytes. The highest hydrophobicity was found for azathioprine (*R*_Mw_ = 1.41; log *k*_w C18_ = 1.34), while 6-thioguanine had the lowest affinity for C18 stationary phase (*R*_Mw_ = 0.21; log *k*_w C18_ = 0.32). The corresponding linear correlation between chromatographic hydrophobicity parameters featured *r* = 0.93. Although limited data on hydrophobicity of immunosuppressants are available in literature, it should be pointed out that comparable data were obtained by various techniques [42,43,44].

#### 2.1.2. Phospholipid Binding Assay

As already reported in literature, the octadecylsilane stationary phase does not resemble a biomembrane phospholipid bilayer and consequently does not fully capture the complex and dynamic nature of human intestinal absorption of drugs [45]. Immobilized artificial membrane (IAM) columns, on the other hand, have shown satisfying performance regarding this matter [46]. In an attempt to gain insight into interaction between azathioprine, 6-mercaptopurine, 6-thioguanine, folic acid and cell membrane, IAM chromatographic column with a monolayer of phosphatidylcholine covalently bound to a propylamino-silica core was used. IAM column permitted the use of phosphate-buffered saline without addition of organic modifier, leading to directly measured log *k*_w IAM_ values and reducing the time of analysis considerably. All values of log *k*_w IAM_ are the average of three measurements, with RSD values lower than 4.1%. Possible occurrence of retention changes due to column aging was monitored by checking the retention times of test compound (mesalazine). During the study the retention time of test compound did not change markedly (RSD = 4.3%), and no correction was done to the retention time experimentally determined for the analytes.

Generally, investigated compounds have shown lower affinity for IAM than for C18 stationary phase (Table 1). The obtained values were in the range from −0.26 (folic acid) to 0.68 (azathioprine). The lack of significant correlation between log *k*_w IAM_ and log *k*_w C18_ values (*r* = 0.61) as well as log *k*_w IAM_ and *R*_Mw_ values (*r* = 0.85) confirms that partition in phospholipids encodes not only hydrophobic intermolecular recognition forces but also ionic bonds, due to electrostatic interactions between electrically charged species and phospholipids. The highest difference between log *k*_w C18_ and log *k*_w IAM_ values (1.25) was found for folic acid, the only compound with two carboxylic groups on the L-glutamate part of the molecule. A possible explanation for the higher affinity of neutral forms of azathioprine, 6-mercaptopurine and 6-thioguanine for IAM surface compared to negatively charged folic acid at pH 7.4 is that repulsive forces between two carboxylic groups and IAM phosphates have occurred.

#### 2.1.3. Protein Binding Assays

As co-administered drugs may exert a competition for the same binding site on plasma proteins, and this competition can have potential clinical consequences, it is of essential importance to evaluate interaction of drugs with plasma proteins in early phases of FDC development. Chromatographic columns with HSA- and AGP-coated stationary phases have been initially developed for chiral separation. Several studies have shown that the retention times of compounds on these phases are proportional to the dynamic distribution constants of the compound between the mobile and stationary phases. It follows that these two biomimetic columns may be used in drug-plasma protein binding studies [45,47,48]. To obtain data suitable for comparison with binding data obtained by other commonly used techniques as well as for inter-laboratory comparison, a standardization and validation of biomimetic chromatographic methods should be done using a set of compounds for which the percentage of binding data is available in literature. In this study, the gradient retention times are standardized using a calibration set of compounds with known percentage of plasma protein binding data (PPB_lit_). The known PPB_lit_ values were converted to the affinity constant, log *K*_PPB_ using Equation (5):

(5) $$\log K_{\mathrm{PPB}}=\log\frac{{\mathrm{PPB}}_{\mathrm{lit}}}{101-{\mathrm{PPB}}_{\mathrm{lit}}}.$$

Acceptable correlations were found between the literature plasma protein binding data and the experimental binding data expressed as gradient retention times (*t*_G_) for the validation set of compounds, with Equations (6) and (7) describing the dependence of log *K* values on *t*_G_ for HSA and AGP columns, respectively:

(6) $$\begin{array}{c} \log K_{\mathrm{HSA}}=1.455\;\left(\pm 0.246\right)\;\log\;t_{G\;\mathrm{HSA}}\;+\;0.172\;\left(\pm 0.120\right)\\ n\;=\;10,\;r\;=\;0.98,\;s_{E}\;=\;0.159 \end{array}$$

(7) $$\begin{array}{c} \log K_{\mathrm{AGP}}=\;5.004\;\left(\pm 1.384\right)\;\log\;t_{G\;\mathrm{AGP}}\;-\;0.964\;\left(\pm 0.350\right)\\ n\;=\;10,\;r\;=\;0.95,\;s_{E}\;=\;0.278, \end{array}$$

where the values in brackets represent 95% confidence intervals, while *s*_E_ represents the standard error. Furthermore, using the slope and intercept values from the calibration line, the logarithmic retention times were converted to log *K*_PPB_ values that can be converted to PPB_HSA_ and PPB_AGP_ of investigated compounds using Equation (8):

(8) $${\mathrm{PPB}}_{\exp}=\frac{101\;\times \;10^{\log K_{\mathrm{PPB}}}}{1\;+\;10^{\log K_{\mathrm{PPB}}}}.$$

Validation of the methods was performed by evaluation of retention of warfarin enantiomers. As retention time on HSA and AGP columns is dependent on the injected amount of compound, the smallest possible amount of sample was injected so that the compound would not saturate the specific binding site. Wide peaks with tailing were observed on both protein stationary phases using concentrations of test compound higher than 10 mg/L. Possible occurrence of retention changes due to column aging was monitored by checking the retention times of selected test compound. During the study no retention value changed more than 0.63% (HSA column) and 0.28% (AGP column).

HSA is the major plasma protein with vital functions acting as depot and carrier for many drugs, and it is also found to be considerably distributed in the interstitial fluid of body tissues. It is well known that HSA binds neutral and negatively charged compounds more strongly than positively charged ones. Thus, the highest binding affinity was found for folic acid (69.40%), the only investigated compound that is, due to fully ionized glutamate carboxyl groups, present in a form of negatively charged folate at the physiological pH (Table 1). Relatively high affinity for HSA was found for lipophilic azathioprine (log *k*_w IAM_ = 0.68, PPB_HSA_ = 49.22%), while 6-mercaptopurine (log *k*_w IAM_ = −0.19, PPB_HSA_ = 20.08%) and 6-thioguanine (log *k*_w IAM_ = 0.03, PPB_HSA_ = 24.11%) bound to HSA to a lesser extent. The obtained results suggest that lipophilic forces are generally predominant in the immunosuppressive drugs–HSA binding mechanism.

Compared to HSA, AGP has a lower plasma concentration in humans. Still, the concentration of AGP in plasma can significantly increase in various conditions, such as inflammatory diseases. All immunosuppressants have shown greater extent of binding to AGP than folic acid (at least 18.7% higher) (Table 1). Given that basic drugs have been shown to bind preferentially to AGP, a better understanding of interaction between this protein and immunosuppressants is valuable. As in the case of HSA-binding mechanism, lipophilic azathioprine showed the highest percentage of AGP binding (log *k*_w IAM_ = 0.68, PPB_AGP_ = 75.96%), followed by 6-thioguanine (log *k*_w IAM_ = 0.03, PPB_AGP_ = 45.96%) and 6-mercaptopurine (log *k*_w IAM_ = −0.19, PPB_AGP_ = 22.15%). The noticeable difference in AGP binding between structurally similar 6-thioguanine and 6-mercaptopurine is likely due to the presence of the basic amino group at position C2 of 6-thioguanine.

#### 2.1.4. Comparison of Biomimetic Chromatographic and Computational Data

In silico based pharmacokinetic modeling approaches have been widely used in drug development processes to provide a fast, low-cost and preliminary screening of compounds prior to in vitro testing [45,49,50]. To perform a comprehensive evaluation of in silico potential in drug development, a number of compounds should be investigated. Still, due to limited number of immunosuppressants used in treatment of IBD available at market, as well as therapeutically reasonable candidates for FDC, a limited number of compounds was included in this study. The collected biomimetic chromatographic data were compared to selected physico-chemical and pharmacokinetic properties obtained by 15 established computational medicinal chemistry methods. Observed parameters included partition coefficient, human intestinal absorption, human colorectal carcinoma cells (Caco-2) permeability and plasma protein binding. The list of theoretical approaches and calculated parameters is given in Table 2. 

In every respect, noticeable discrepancies among theoretical partition coefficients were observed. Intercorrelation between the data gave correlation coefficients in the range from 0.01 to 0.99. Average calculated log *P* values were in the range from −0.84 to 1.22. It is obvious that some of calculation procedures overestimate log *P* values of investigated compounds, while others underestimate them. Still, it can be found that MlogP values correlate well with both hydrophobic chromatographic parameters featuring *r* = 0.93 with *R*_Mw_ and *r* = 0.99 with log *k*_w C18_. As mentioned above, the interaction of investigated compounds with membrane phospholipids is complex and governed not only by hydrophobicity but also electrostatic interactions, especially in the case of negatively charged folic acid. Therefore, it is worth pointing out that the linear correlation between log *k*_w IAM_ and log *P* values calculated by programs Mcule and Chemicalize is noteworthy featuring *r* ≥ 0.94, while correlations with other predicted log *P* values were lower (0.01 ≤ *r* ≤ 0.85).

The obtained results might indicate that theoretical approaches of the two above-mentioned programs are powerful enough to predict affinity of investigated compounds for immobilized phosphatidylcholine. 

Finally, it is relevant to evaluate whether IAM, HSA and AGP biomimetic chromatographic columns might be comparable with in silico pharmacokinetic profiling of selected drugs. As uptake of folic acid is mediated by transport proteins, it was excluded from evaluation of passive absorption by IAM chromatography. Evaluation of all indices describing drug absorption indicates that the majority of the programs predict somewhat higher absorption rates for 6-mercaptopurine and 6-thioguanine compared to more lipophilic azathioprine. This might be related to their smaller size and thus easier penetration through the cell membrane. It might be concluded that passive absorption of the drug through the gastrointestinal barrier is a very heterogeneous process that cannot be accounted for via simple affinity for immobilized phosphatidylcholine, but other properties, such as the size of the molecule, should also be considered.

The trend of increasing affinity for immobilized human serum albumin column in order 6-mercaptopurine < 6-thioguanine < azathioprine < folic acid was in accordance with both parameters describing plasma protein binding calculated by ADMETLab and pkCSM programs. Moreover, it should be emphasized that the linear correlation between PPB_HSA_ values and computationally predicted values featured *r* values higher than 0.94. On the other hand, experimental AGP binding data did not relate well to any of predicted parameters (*r* ≤ 0.52).

It should be pointed out that none of calculation procedures clearly state which plasma protein is involved in distribution of investigated compounds, which leaves room for considerable improvement of theoretical approaches.

### 2.2. Analysis of Pharmaceutical Dosage Forms

#### 2.2.1. Method Development

Due to different physico-chemical properties of immunosuppressants and folic acid, as well as high dose differential between APIs combined in three proposed FDCs (5 mg of folic acid combined with 40 mg of 6-thioguanine or 50 mg of azathioprine or 50 mg of 6-mercaptopurine per dosage form), developing a unique sample preparation procedure and simultaneous chromatographic method for their determination in FDC was quite a challenging task. Optimization of the experimental conditions was split into two major areas. First, simultaneous chromatographic method for determination of active ingredients in FDC was optimized. Afterwards the proposed method was used for optimization of sample preparation procedure.

In the light of available individual methods for the determination of azathioprine, 6-mercaptopurine, 6-thioguanine and folic acid, different chromatographic parameters were tested to establish the most suitable chromatographic conditions for their simultaneous determination in FDCs [20,23,32]. Parameters such as composition of stationary and mobile phases as well as temperature of analysis were systematically studied so as to achieve good resolution of adjacent peaks, symmetric peaks, high column performance and acceptable runtime. Afterwards, extraction solvent and time were optimized to attain high extraction efficiency.

A type of chosen chromatographic column had a significant influence on separation of compounds. Due to considerable differences in polarity of analytes, a compromise between hydrophilic and lipophilic character of sorbents had to be found [20,23,32,33]. Therefore, three kinds of chromatographic columns were investigated: XBridge C18 (150 × 4.6 mm, particle size 5 µm) by Waters (Milford, MA, USA), XBridge Phenyl (150 × 4.6 mm, particle size 5 µm) by Waters (Milford, MA, USA) and Zorbax SB C8 (150 × 4.6 mm, particle size 5 µm) by Agilent Technologies (Santa Clara, CA, USA). The baseline separation of critical pair of analytes, 6-thioguanine and 6-mercaptopurine, was achieved only using Zorbax SB C8 column, which was shown to be the most suitable for the separation of analytes in a single run and this column was used for all further experiments.

The composition of mobile phase appeared to be another critical factor in achieving the appropriate chromatographic behavior and satisfying separation of analytes. The selection of acetonitrile as organic modifier, as opposed to methanol, was based primarily on lower viscosity and, accordingly, lower operating pressure. It was found that pH of mobile phase had a profound effect on the ionization of analytes and the resultant chromatographic behavior, especially in the case of folic acid. The p*K*a’s of two carboxylic groups on the L-glutamate part of folic acid are 3.5 and 4.8, respectively [33,40]. Therefore, under reversed phase conditions an acidified mobile phase was required to improve retention of folic acid. To optimize retention as well as peak shape of folic acid, discrete levels (0.05% to 0.2%) of a volatile organic acid (acetic and formic acid) were added to the mobile phase. Peak tailing was noted with both investigated organic acids’ concentrations of less than 0.1% (peak asymmetry factor was greater than 1.2). Addition of both organic acids to mobile phase at levels 0.1% and 0.2% was found to give symmetric peaks, higher column performance and good resolution among all four analytes. Mobile phase with 0.1% of formic acid was selected for further investigations by considering the working life of the column. Initially, isocratic mode of separation was tested and was found insufficient to resolve all four analytes with good peak shape characteristics. Complete separation was achieved by conversion of the isocratic elution to a gradient mode. Moreover, the optimized gradient program dramatically facilitated achievement of shorter runtime (all analytes were eluted within 7 min).

**Figure 2:**
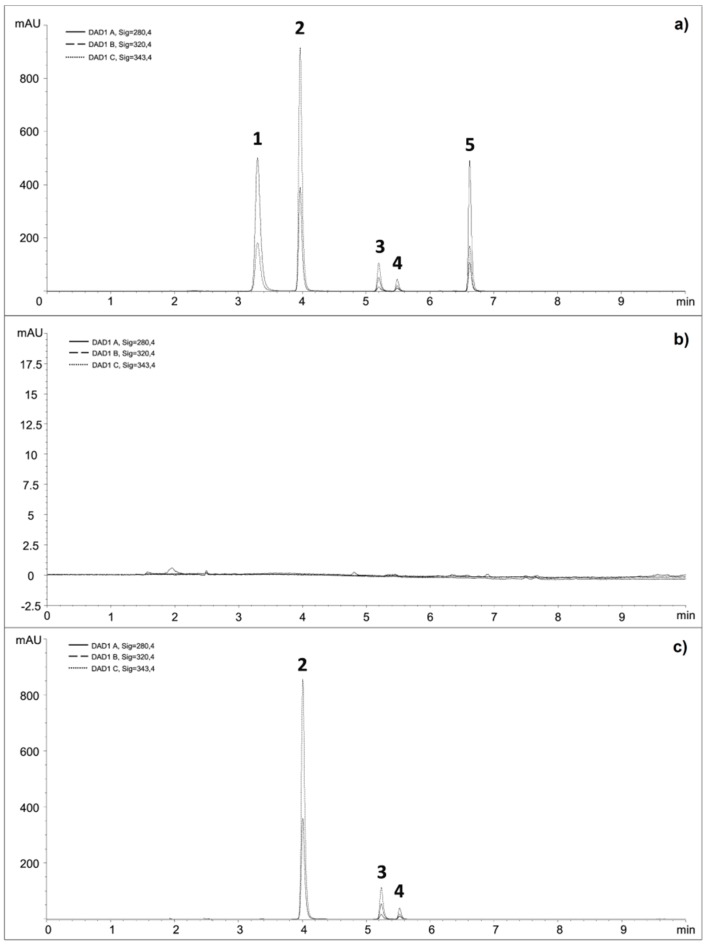
Chromatograms of: (**a**) mixed standard solution of 6-thioguanine (1), 6-mercaptopurine (2), internal standard (3), folic acid (4) and azathioprine (5); (**b**) 0.02 M NaOH solution containing commonly used excipients; (**c**) proposed fixed-dose combination (FDC) containing 6-mercaptopurine (50 mg/L) (2) and folic acid (5 mg/L) (4) with the addition of internal standard (3).

As the column temperature may affect the efficiency of chromatographic separation, the impact of three different temperatures (20, 25 and 30 °C) using the retention and resolution factors as the basic criteria was evaluated. Inadequate peak symmetry (less than 0.7) was observed at 20 °C. The separation was markedly faster at higher temperatures, but seeing that 6-thioguanine eluted considerably earlier, resolution between 6-thioguanine and the adjacent peaks of excipients became critical. Thus, the temperature of 25 °C was selected as the optimal temperature. Different compounds (6-methylthioguanine, methotrexate and caffeine) were assessed to be used as an internal standard. Among these compounds, 6-methylthioguanine showed good resolution and instrumental response with all APIs included in three proposed FDCs and was therefore selected as the internal standard. A chromatogram of mix standard solution separated by optimized chromatographic method is shown in Figure 2A. 

Differences in chemical properties of the studied compounds hindered the simultaneous extraction of all four compounds using one set of extraction conditions. Protocols utilizing aqueous or organic solvent extraction solutions (acidic, alkaline or neutral pH) combined with at ambient and elevated temperatures shaking and sonication procedures have all been reported [51]. Therefore, one-variable-at-a-time method was applied to find a ubiquitous method suitable for all investigated analytes. Preliminary investigations have shown that methanol improved extraction of 6-thioguanine, 6-mercaptopurine and azathioprine (it was found to be more than 5.7% effective in methanol than in ultrapure water), while folic acid was found slightly soluble in organic solvents (extraction efficiency in methanol was only 4.6%) as well as in water (extraction efficiency was 22.4%), but soluble in basic aqueous solutions. Therefore, a basic solution with NaOH was selected for further investigation. Evaluation of optimal concentrations of NaOH (0.02 M, 0.05 M and 0.1 M) showed that extraction of all analytes has been improved in 0.02 M NaOH, while degradation of azathioprine was observed at higher levels of NaOH. Further, the investigation of ultrasonication time (up to 120 min) indicated that higher extraction rates (more than 96.1%) and better precisions (RSD lower than 3.3%) for all compounds were achieved when the time was 15 min or longer. Folic acid in solution is known to decompose when exposed to ultraviolet light and/or increased temperature; thus, all experiments were performed at ambient temperature using amber laboratory glassware [52].

#### 2.2.2. Validation of the Method

The newly developed method was validated according to the ICH guidelines with respect to selectivity, linearity, sensitivity, precision, accuracy, stability and robustness [53]. All validation parameters were evaluated using placebo solution spiked with standard solutions.

As an integral part of the analytical method, system suitability test was used to verify adequacy of the resolution and reproducibility of the chromatographic system. It was established by analysis of seven replicate injections of mixed standard (100% level of concentration in proposed FDCs) solution and parameters such as retention time, relative retention time, resolution, capacity factor, number of theoretical plates, tailing factor and peak area of the analytes were calculated. The results of system suitability are shown in Table 3.

The selectivity of the developed method was evaluated by injecting a mixture of standard solutions and was assessed by peak purity test (comparison between analyte peak and auto threshold in the purity plot). The peaks of analyte of interest were not found to be attributed to more than one component, indicating the method to be selective (peak purity factors were higher than 999.8). Afterwards, a 0.02 M solution containing excipients chosen as the ones used commonly in tablet formulations was analyzed. The majority of the excipients were eluted from the column within the first 2.5 min, which indicates that the excipients were not interfering with the assay (Figure 2B). Finally, mix standard solution containing excipients chosen as the ones used commonly in tablet formulations was analyzed. The obtained peak purity factors were also higher than 999.8.

The linearity of the method was evaluated by analyzing at least six freshly prepared solutions, in the concentration range between 0.2 and 20 mg/L for folic acid, 5 and 80 mg/L for 6-thioguanine and 5 and 100 mg/L for azathioprine and 6-mercaptopurine (Table 4). Calibration curves showed linear responses for all analytes over dynamic ranges, and the corresponding regression correlation coefficients (*r*) were all above 0.999. Due to a high dose differential between APIs combined in proposed FDCs, limits of detection (LOD) and quantitation (LOQ) of the method were one of the most important validation parameters. LOD and LOQ, based on 3 and 10 times the signal-to-noise ratio, respectively, were determined by repeated injections of diluted standard solutions. According to data presented in Table 4, satisfying linearity range as well as sensitivity was obtained for all four analytes.

Precision studies were done by evaluation of repeatability and intermediate precision. Intra-day studies were done by injecting the solutions at one concentration level (100% level of concentration in proposed FDCs) six different times on the same day. Inter-day studies were done in triplicate every day up to three consecutive days. Low values of RSD (%) showed that the method is precise within the acceptance limit of ±2%. The intra- and inter-day variability or precision data are given in Table 5. The results indicate good precision of the developed method.

The proposed method was evaluated for its accuracy by the analysis of the placebo solutions spiked with three different standard concentration levels (80%, 100% and 120% level of concentration in proposed FDCs). As it can be seen in Table 5, the calculated accuracy was always within the acceptance limit of ±3% of the nominal concentration.

The stability of APIs’ sample solutions was evaluated by keeping them in tightly closed amber vials on the laboratory worktable at room temperature for 2 h and in a rack on the autosampler at 4 °C for 24 h. Furthermore, long-term stability was evaluated in the freezer at −20 °C for 30 days. The recoveries were in the range from 96.4% to 103.3%, which indicates that investigated compounds were stable in described experimental conditions within the given periods. 

The impact of different chromatographic parameters on the peak areas and peak shapes of all APIs was examined by small deliberate changes in the mobile phase composition (±1%) and flow rate (±10%) as well as temperature (± 1 °C) to establish the robustness of the proposed method. In all deliberately varied conditions, the RSD of peak areas of all APIs were found to be well within the acceptable limit of 5%. The tailing factor for all peaks was found to be lower than 1.5.

#### 2.2.3. Application of the Method

The developed method was successfully applied for analysis of azathioprine, 6-mercaptopurine, 6-thioguanine and folic acid in marketed tablet formulation. Firstly, 20 tablets of each pharmaceutical product were weighed individually; the RSDs of the tablet weights were lower than 3.0%, indicating satisfying weight uniformity. Upon analysis of the samples, no interferences of excipients were observed as peak purity factors were higher than 999.7. The amounts recovered were expressed as a percentage of the label claim, and obtained results were in the range between 96.1% and 104.4% (Table 6). Contents of all of the analyzed samples conform to the United States Pharmacopoeia [54] and British Pharmacopoeia regulations [55].

Afterwards, the method was successfully applied to analysis of all three proposed FDCs. A representative chromatogram for analysis of proposed FDC containing 6-mercaptopurine and folic acid is shown in Figure 2C.

## 3. Materials and Methods 

### 3.1. Reagents and Chemicals

Standards, certified reference material, for calibration and validation of biomimetic columns were: Acetylsalicylic acid, caffeine, cefalexin, chloramphenicol, cimetidine, codeine hydrochloride dihydrate, furosemide, ibuprofen, mesalazine, metronidazole, nifedipine, paracetamol, propranolol hydrochloride, salicylic acid, sulfadiazine, sulfamethoxazole, thiamine hydrochloride and warfarin purchased from Sigma-Aldrich (St. Louis, MO, USA). Azathioprine; folic acid; 6-mercaptopurine and 6-thioguanine, United States Pharmacopeia reference standard and 6-methylthioguanine (≥95%) have also been obtained from Sigma-Aldrich. Acetonitrile, ethanol, iso-propanol, methanol, formic acid, all HPLC grade; sodium hydroxide; ACS reagent (97.0%) pellets; sodium nitrate (≥99.0); phosphate-buffered saline tablets (0.01 M, 0.0027 M potassium chloride and 0.137 M sodium chloride, pH 7.4 at 25 °C); potassium phosphate monobasic and potassium phosphate dibasic (HPLC grade) were provided by Sigma-Aldrich. Ultra-pure water was obtained using a MilliQ UF-Plus system (Millipore, Darmstadt, Germany); resistivity MΩcm^−1^ >18 at 25 °C and TOC <5 ppb. The excipients included hydroxypropyl methylcellulose Methocel K100M Premium CR (Colorcon, Harleysville, PA, USA), magnesium stearate (Acros Organics, Princeton, NJ, USA), lactose monohydrate, stearic acid, wheat, rice and corn starch (Kemig, Zagreb, Croatia).

### 3.2. Equipment

All assays were performed on an Agilent 1100 series HPLC system (Agilent Technologies, Waldbronn, Germany) consisting of a quaternary pump, autosampler, vacuum degasser, column compartment and DAD. Data acquisition and processing were carried out using ChemStation for LC 3D software (version rev A.10.02[1757]).

Evaluation of TLC plates was done using Camag UV Cabinet 4 consisting of a UV Lamp 4 (dual wavelength 254/366 nm) and a Viewing Box 4 (Camag, Muttenz, Switzerland), while Elmasonic Ultrasonic BathModel XtraTT with heat and time controller (Elma Schmidbauer, Singen, Germany) was employed for all ultrasonic extractions. In sample preparation step centrifuge model Z 326 K by Hermle (Wehingen, Germany) with digital time, temperature and speed control were also used. Weighing measurements were performed using MX5 Microbalance (Mettler-Toledo, Greifensee, Switzerland) with 1 µg readability.

### 3.3. Preparation of Stock and Working Solutions

Solutions containing 100 mg/L of standards used for calibration and validation of biomimetic columns were dissolved in methanol. For the simultaneous determination method, stock solutions containing 200 mg/L of azathioprine, 6-mercaptopurine and 6-thioguanine were prepared by dissolving accurately weighed amounts of reference standards in diluent 1 (containing methanol and 0.1 M NaOH, 95:5, *v/v*), while stock solution containing 200 mg/L of folic acid was prepared by dissolving accurately weighed amount of reference standard in diluent 2 (containing methanol, ultrapure water and 0.1 M NaOH, 70:29:1, *v/v/v*). Working solutions of lower concentrations were freshly prepared by dilution with water. Internal standard, 6-methylthioguanine (200 mg/L in diluent 1) was added to each working solution to make its concentration 10 mg/L in each sample. For the validation study, placebo solution in 0.02 M NaOH containing mixture of the most commonly used excipients was used. The drug to excipient ratio used was similar to that in commercial formulations. All solutions were stored at 4 °C in amber glassware. Prior to use, all solutions were filtered through 0.2 μm polyethersulfone filters (Obrnuta faza, Pazin, Croatia).

### 3.4. RP-TLC Assay

TLC experiments were performed on commercially available 10 × 10 cm RP-18 TLC plates (Merck, Darmstadt, Germany). Methanol-phosphate-buffered saline mixtures were used as mobile phases. The methanol volume fraction was varied from 20% to 80% in 5% steps. A sufficient amount of mobile phase was placed in a Camag flat-bottom chamber for 10 × 10 cm plates with a stainless-steel lid to have a level of 5 mm. After saturation of chambers with solvent vapor for 30 min, the plates were developed to a distance of 9 cm at room temperature, dried in open air for 5 min and visualized under λ = 254 nm UV light.

### 3.5. RP-HPLC Assay

Hydrophobicity of the compounds was also studied on a reversed-phase Symmetry C18 column (150 × 4.6 mm, 3.5 µm particle size) obtained by Waters (Milford, MA, USA). Methanol-phosphate-buffered saline mixtures were used as mobile phases. The methanol volume fraction was varied from 10% to 50% in 5% steps. Each mobile phase was shaken vigorously, filtrated through cellulose nitrate filter (0.45 μm, Sartorius, Goettingen, Germany) and degassed by sonication (5 min) before use. Injection volume was set at 10 µL, and the measurements were carried out at 25.0 ± 0.1 °C and at flow rate 1.0 mL/min. The absorbance of the analytes during a chromatographic run was collected in the spectral range 200–400 nm, and the detection wavelength for each analyte was the one providing the maximum peak height.

### 3.6. Phospholipid Binding Assay

The binding of compounds to the immobilized artificial membrane (IAM) was measured using commercially available immobilized phosphatidylcholine columns (IAM.PC.DD2, 100 x 4.6 mm, 300 Å particle size) obtained by Regis Technologies (Morton Grove, IL, USA). The mobile phase consisted of phosphate-buffered saline that was filtrated through cellulose nitrate filter and degassed (5 min) by sonication before use. Injection volume was set at 10 µL and the measurements were carried out at 25.0 ± 0.1 °C and at flow rate 1.0 mL/min. The absorbance of the analytes during a chromatographic run was collected in the spectral range 200–400 nm and the detection wavelength for each analyte was the one providing the maximum peak height.

### 3.7. Protein Binding Assays

The interactions of analytes with plasma proteins human serum albumin (HSA) and α1-acid glycoprotein (AGP) have been measured using commercially available chemically bonded HSA (Chiralpak-HSA, 50 × 4.6 mm, 5 μm particle size) and AGP (Chiralpak-AGP, 50 × 4.6 mm, 5 μm particle size) columns obtained by ChromTech (Cedex, France). The mobile phase consisted of 20 mM potassium phosphate buffer with the pH adjusted to 7.0 (A) and iso-propanol (B). The gradient program was as follows: 0–3 min, linear gradient 0–30% B, 3–10 min, isocratic 30% B. The total run time was 15 min to allow re-equilibration of the protein phase with the buffer. Injection volume was set at 10 µL, and the measurements were carried out at 25.0 ± 0.1 °C and at flow rate 1.5 mL/min. The absorbance of the analytes during a chromatographic run was collected at the following wavelengths: 250, 280, 300, 320 and 340 nm.

### 3.8. Analysis of Pharmaceutical Dosage Forms

#### 3.8.1. Sample Preparation

Twenty randomly chosen tablets were weighed, and the average weight of the tablet was determined. All tablets were crushed and powdered. For the analysis of the individual samples, an amount of powdered tablets equivalent to 0.2 mg of folic acid, 1.6 mg of 6-thioguanine, 2 mg of azathioprine or 2 mg of 6-mercaptopurine was weighed. Regarding analysis of three proposed FDCs, an adequate amount of prepared powders, equivalent to the proposed therapeutic dose ratio of each FDC (0.2 mg of folic acid combined with 1.6 mg of 6-thioguanine or 2 mg of azathioprine or 2 mg of 6-mercaptopurine) was weighed and mixed. For both of the procedures, the powders were transferred into 10 mL amber volumetric flasks and dispersed in 0.02 M NaOH with addition of internal standard solution. It was then followed by sonication for 15 min to provide complete dissolution. The final volume was adjusted with ultrapure water and the mixture was centrifuged at 6000 rpm for 10 min to separate the supernatant from non-dissolved excipients. Afterwards, the clear supernatant liquor was filtrated through a 0.2 µm polyethersulfone injection filter, appropriately diluted and transferred into an amber HPLC vial.

#### 3.8.2. Sample Analysis

Chromatographic separation was achieved using a Zorbax SB C8 column (150 × 4.6 mm, 5 μm particle size) obtained by Agilent Technologies. A Zorbax SB C8 guard column (10 × 4.6 mm, 5 μm particle size) was also utilized. The mobile phase consisted of water (eluent A) and acetonitrile (eluent B), both acidified with 0.1% (*v/v*) formic acid. Each component of the mobile phase was filtrated through a cellulose nitrate filter and degassed before use in an ultrasonic bath for 5 min. The elution was a two-step gradient program with flow of 1.0 mL/min throughout the method: 0–3 min, linear gradient 5–20% B, 3–10 min linear gradient 20–70% B. Total run time was 15 min to allow re-equilibration of the stationary phase for the following analysis. Injection volume was set at 10 µL and a mixture of acetonitrile and ultrapure water (80:20, *v/v*) was used as needle wash solvent. The measurements were carried out at 25.0 ± 0.1 °C. The absorbance of the analytes during a chromatographic run was collected in the spectral range 200–400 nm and the detection wavelength for each analyte was the one providing the maximum peak height.

## 4. Conclusions

In this work, new FDCs of thiopurine immunosuppressants and folic acid are proposed. Two key prerequisites in new FDC development, pharmacokinetic profiling and simultaneous determination of APIs, have been conducted.

Evaluation of pharmacokinetic profiles of azathioprine, 6-mercaptopurine, 6-thioguanine and folic acid was performed using different chromatographic techniques and in silico approach.

The simultaneous determination method is capable of resolving and quantifying four analytes in a relatively short run time, with a simple and time-efficient sample preparation. Moreover, most commonly used excipients do not interfere with the determination of the analytes, demonstrating the selectivity of the method. The number of APIs found was in agreement with the label claim of commercially available formulations, proving the method to be reliable. The method shows potential to be used in testing of content uniformity and dissolution profiling in the formulation process.

However, proposed FDCs need to be evaluated further in terms of stability of multi-drug combination versus single API dosage forms by means of stress studies and stability-indicating methods, which will be our further goals.

## Figures and Tables

**Table 1:** Experimental parameters obtained by chromatographic techniques.

Analyte	*R*_Mw_^1^	log *k*_w C18_^2^	log *k*_w IAM_^3^	PPB_HSA_^4^ (%)	PPB_AGP_^5^ (%)
6-thioguanine	0.21	0.32	0.03	24.11	45.96
6-mercaptopurine	0.23	0.45	−0.19	20.08	22.15
Folic acid	0.57	0.99	−0.26	69.40	3.45
Azathioprine	1.41	1.34	0.68	49.22	75.96

^1^*R*_Mw_—chromatographic parameter of hydrophobicity obtained by RP-TLC; ^2^ log *k*_w C18_—chromatographic parameter of hydrophobicity obtained by RP-HPLC; ^3^ log *k*_w IAM_—chromatographic parameter of lipophilicity obtained by IAM-HPLC; ^4^ PPB_HSA_—percentage of analyte bound to human serum albumin (HSA) obtained by HSA-HPLC; ^5^ PPB_AGP_—percentage of analyte bound to *α*1-acid glycoprotein (AGP) obtained by AGP-HPLC.

**Table 2:** List of calculated physico-chemical and pharmacokinetic parameters.

Parameter	Program	6-thioguanine	6-mercaptopurine	Folic Acid	Azathioprine
partition coefficient	ACDLabs	−0.12	−0.18	−2.48	0.67
	ADMETLab	0.60	1.02	−0.05	1.15
	ALOGPS	−0.36	−0.13	−0.04	0.84
	ChemExper	−0.02	0.43	−1.43	0.42
	Chemicalize	−0.35	−0.12	−0.69	1.17
	Mcule	1.18	1.02	1.00	1.67
	MlogP	−1.31	−1.16	−0.62	−0.26
	Molinspiration	−0.58	−0.39	−2.37	0.50
	Molsoft	−0.36	0.23	−0.95	0.10
	pkCSM	0.60	1.02	−0.04	1.15
	PreADMET	−0.35	−0.47	0.65	0.67
	Silicos-it	2.05	2.78	0.24	−1.11
	SwissADME	0.28	0.62	−0.42	0.04
	XlogP	−0.07	0.70	−1.08	0.10
human intestinal absorption (%)	ADMETLab	76.7	81.3	39.8	78.2
	pkCSM	91.0	91.0	2.3	78.6
	PreADMET	83.1	87.8	23.2	75.5
Caco-2 permeability [log(cm × 10^−6^/s)]	ADMETLab	0.82	1.53	−0.33	1.41
	admetSAR	0.49	0.62	−1.07	0.11
	pkCSM	0.17	1.14	−0.98	0.62
	PreADMET	0.29	0.28	0.31	−1.38
plasma protein binding (%)	ADMETLab	27.0	24.2	67.9	35.8
	pkCSM	32.2	28.1	63.9	60.4

**Table 3:** System suitability data.

Analyte	Parameter (Mean Value ± Standard Deviation), *n* = 7						
	*t*_R_^1^	*Rt*_R_^2^	*Rs* ^3^	*K* ^4^	N ^5^	TF ^6^	A ^7^
6-thioguanine	3.29 ± 0.01	0.635 ± 0.001	/	1.06 ± 0.01	8716 ± 198	1.38 ± 0.02	2871.6 ± 27.8
6-mercaptopurine	3.96 ± 0.01	0.761 ± 0.001	5.69 ± 0.05	1.48 ± 0.01	28462 ± 330	1.35 ± 0.02	3522.4 ± 35.5
6-methylthioguanine	5.20 ± 0.01	/	13.57 ± 0.31	2.25 ± 0.01	53336 ± 1169	1.34 ± 0.01	388.2 ± 2.8
Folic acid	5.49 ± 0.01	1.055 ± 0.001	3.37 ± 0.03	2.43 ± 0.01	75297 ± 1956	1.28 ± 0.01	141.8 ± 1.7
Azathioprine	6.63 ± 0.02	1.275 ± 0.001	14.22 ± 0.07	3.14 ± 0.01	110071 ± 2807	1.34 ± 0.02	1578.5 ± 16.8

^1^*t*_R_—retention time; ^2^*Rt*_R_—relative retention time; ^3^*Rs*—resolution; ^4^*k*—capacity factor; ^5^ N—theoretical plates; ^6^ TF—USP tailing factor; ^7^ A—peak area; /—not applicable.

**Table 4:** Method calibration data.

Analyte	Linearity Range (mg/L)	Equation	*r* ^1^	*s*_E_^2^	LOD (mg/L)	LOQ (mg/L)
6-thioguanine	5–80	*y* = 0.251 *x* − 0.132	0.9996	0.124	0.1	0.2
6-mercaptopurine	5–100	*y* = 0.230 *x* − 0.034	0.9997	0.140	0.1	0.2
Folic acid	0.2–20	*y* = 0.081 *x* − 0.007	0.9999	0.013	0.1	0.2
Azathioprine	5–100	*y* = 0.102 *x* + 0.016	0.9998	0.048	0.1	0.2

^1^*r*—Pearson correlation coefficient; ^2^*s*_E_—standard error.

**Table 5:** Intra- and inter-day assay precision and accuracy data.

Analyte	Precision Relative Standard Deviation (RSD) (%)		Accuracy Recovery ± RSD (%)		
	Intra-Day Precision (*n* = 6)	Inter-Day Precision (*n* = 9)	Low	Medium	High
6-thioguanine	0.73	0.75	99.49 ± 0.80	100.42 ± 1.66	98.79 ± 0.68
6-mercaptopurine	0.81	0.79	100.35 ± 1.74	100.79 ± 0.69	99.01 ± 0.76
Folic acid	1.04	1.11	99.78 ± 0.81	97.57 ± 0.37	100.25 ± 0.87
Azathioprine	0.40	0.64	100.38 ± 0.86	100.38 ± 1.19	99.55 ± 0.58

**Table 6:** Results of analyses of marketed formulations (*n* = 3).

Commercial Formulation	Manufacturer	Active Substance	Amount Labeled (mg)	Amount Found (mg)	Detected/Labeled (%)	RSD (%)
Thiosix^®^	Teva Nederland B.V, Haarlem, the Netherlands	6-thioguanine	10	10.44	104.42	2.90
Puri-Nethol^®^	Aspen, Dublin, Ireland	6-mercaptopurine	50	48.07	96.13	2.89
Folacin^®^	JGL, Rijeka, Croatia	folic acid	5	4.99	99.95	3.31
Imuran^®^	Aspen, Dublin, Ireland	azathioprine	50	51.63	103.26	2.24
